# New Books

**Published:** 2007-08

Acid Rain—Deposition to Recovery

P. Brimblecombe, H. Hara, D. Houle, M. Novak, eds.

New York:Springer, 2007. 420 pp. ISBN: 978-1-4020-5884-4, $169.00

Biosphere Implications of Deep Disposal of Nuclear Waste: The Upwards Migration of Radionuclides in Vegetated Soils

H.S. Wheater, J.N.B. Bell, A.P. Butler, B.M. Jackson, L. Ciciani, D. Ashworth, G.G. Shaw

Hackensack, NJ:World Scientific Publishing Co., 2007. 350 pp. ISBN: 1-86094-743-8, $95.00

Climate Change—Environment and History of the Near East, 2nd ed.

Arie S. Issar, Mattanyah Zohar

New York:Springer, 2007. 290 pp. ISBN: 978-3-540-69851-7, $169.00

Elements of Environmental Chemistry

Ronald A. Hites

Hoboken, NJ:John Wiley & Sons, Inc., 2007. 204 pp. ISBN: 978-0-471-99815-0, $39.95

Endocrine-Disrupting Chemicals: From Basic Research to Clinical Practice

Andrea C. Gore, ed.

New York:Springer, 2007. 300 pp. ISBN: 978-1-58829-830-0, $139.00

Environmental Applications of Nanomaterials: Synthesis, Sorbents and Sensors

Glenn E. Fryxell, Guozhong Cao, eds.

Hackensack, NJ:World Scientific Publishing Co., 2007. 520 pp. ISBN: 1-86094-662-3, $100.00

Environmental Public Health Impacts of Disaster: Hurricane Katrina, Workshop Summary

Lynn Goldman, Christine Coussens

Washington, DC:National Academies Press, 2007. 100 pp. ISBN: 0-309-10500-5, $18.00

Essential Environmental Science

Daniel B. Botkin, Edward A. Keller

Hoboken, NJ:John Wiley & Sons, Inc., 2007. 512 pp. ISBN: 978-0-471-704-11-9, $95.95

Improving the Nation’s Water Security: Opportunities for Research

Committee on Water System Security Research, National Research Council

Washington, DC:National Academies Press, 2007. 170 pp. ISBN: 0-309-10566-8, $36.50

Mine Wastes: Characterization, Treatment and Environmental Impacts, 2nd ed.

Bernd Lottermoser

New York:Springer, 2007. 304 pp. ISBN: 978-3-540-48629-9, $169.00

Models in Environmental Regulatory Decision Making

Committee on Models in the Regulatory Decision Process, National Research Council

Washington, DC:National Academies Press, 2007. 218 pp. ISBN: 0-309-11000-9, $40.27

Our Precarious Habitat … It’s in Your Hands

Melvin A. Bernarde

Hoboken, NJ:John Wiley & Sons, Inc., 2007. 464 pp. ISBN: 978-0-471-74065-0, $42.50

Single Cell Diagnostics: Methods and Protocols

Alan Thornhill, ed.

Totowa, NJ:Humana Press, 2007. 238 pp. ISBN: 978-1-58829-578-1, $99.00

Thirst: Fighting the Corporate Theft of Our Water

Alan Snitow, Deborah Kaufman, Michael Fox

Hoboken, NJ:John Wiley & Sons, Inc., 2007. 304 pp. ISBN: 978-0-7879-8458-8, $27.95

Toxicity Testing in the Twenty-first Century: A Vision and a Strategy

Committee on Toxicity and Assessment of Environmental Agents, National Research Council

Washington, DC:National Academies Press, 2007. 146 pp. ISBN: 0-309-10988-4, $42.00

Water Resources in the Middle East

Hillel Shuval, Hassan Dweik, eds.

New York:Springer, 2007. 458 pp. ISBN: 978-3-540-69508-0, $169.00

